# Pharmacological errors in NICU

**DOI:** 10.1186/1824-7288-40-S2-A53

**Published:** 2014-10-09

**Authors:** Silvia Foligno, Virginia Garofalo, Anna Portanova, Andrea Dotta

**Affiliations:** 1 Neonatal Intensive Care Unit, Department of Medical and Surgical Neonatology, Bambino Gesù Children’s Hospital, IRCCS, Rome, Italy

## Background

Medical errors are particularly frequent in Neonatal Intensive Care Units (NICUs) [1], increasing morbidity and mortality of newborns [2]. This category of patients requires the application of high technology and needs individualized medical prescription mainly based on body weight and gestational age[3]. The most frequent event categories are wrong medication, dose, schedule, or infusion rate; error in administration or method of using a specific treatment; patient misidentification; error or delay in diagnosis and in the performance of an operation, procedure, or test [2]. The staff inexperience and intensity of workload are indicated as risk factors [4]. Most vulnerable newborns are those with indwelling infusion lines and long length of stay [1]. Common errors are due to the dose because of the lack of reference standards and of awareness of pharmacokinetics and pharmacodynamics drug [1]. The Joint Commission for Accreditation of Health Care Organization (JCHAO) estimates as many as 95% of adverse drug reactions (ADRs) in children remain unreported each year[5]. Frequent analysis of reporting data, training and meeting of all participating NICUs, implementation of computerized physician order entry (CPOE), and improve the staff with supervisor pharmacist might be help to detect errors and to learn about these [1,4].

## Materials and methods

We carried out our study from 2011 and 2012 in Department of Medical and Surgical Neonatology of Bambino Gesù Children’s Hospital. We recorded throughout retrospective methods nursing reports to detect an error or incidents. We used voluntary reporting, non punitive, of medical errors by health care providers.

## Results

**Figure 1:**
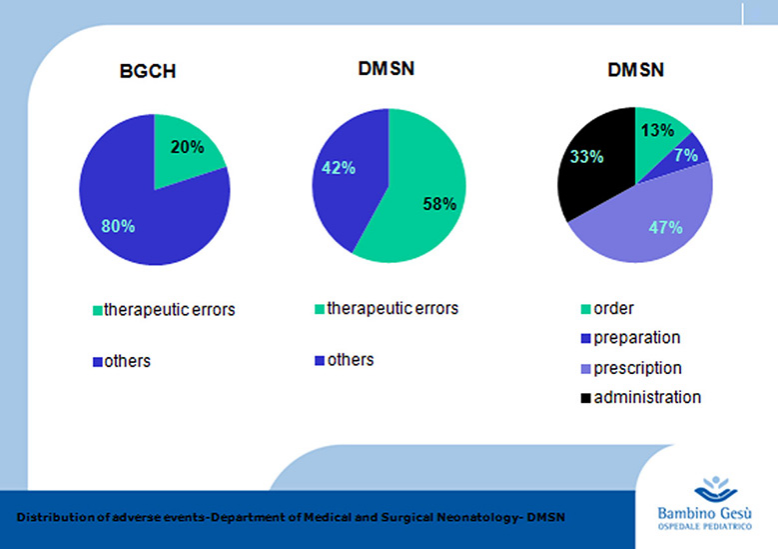
Adverse events at the Bambino Gesù Children’s Hospital from 2011 to 2012. Distribution of adverse events at the Bambino Gesù Children's Hospital (BGCH), at the Department of Medical and Surgical Neonatology (DMSN) and the percentage of different stages of the drug within the DMSN.

From 2011 and 2012 we detected 29 adverse events in Neonatal Department; 15 (58%)of whom were therapeutic errors concerning of drug process: 2 (13%) order, 1 (7%) preparation, 7 (46%) prescription, 5 (33%) administration (Figure 1). While in the Bambino Gesù Children’s Hospital the adverse events related to pharmacological errors were only 20%.

## Conclusions

The voluntary reporting system represents the best option to detect the human errors. In our experienced the introduction of shared protocols, of the nurse staff training, and the following of the JCHAO directives have been achieved to identify all procedures performed for patient care. To reduce the ADRs the Paediatric Investigation Plans should be required by the Paediatric Committed to guarantee safer and tolerated drugs, especially for newborns.
